# Self-Compassion and General Well-Being among Self-Quarantined Residents: Mediation by Certainty in Control and Moderation by Positive Coping

**DOI:** 10.1192/j.eurpsy.2023.740

**Published:** 2023-07-19

**Authors:** A. Li, S. Wang

**Affiliations:** 1 College of Teacher Education, Nanjing University of Information Science & Technology; 2 Department of Mental Health Education, Nanjing Audit University, Nanjing, China

## Abstract

**Introduction:**

The outbreak of the Coronavirus Disease 2019 (COVID-19) has caused adverse outcomes on tens of millions of people worldwide, both physically and psychologically. As a public health response, quarantine has been recruited as a national measure in COVID-19, which subjects people who are suspected and confirmed cases to strictly isolation. Unfortunately, people may suffer from various adverse effects under self-quarantine at home. Thus, it is crucial to explore how to improve the psychological outcomes of self-quarantined residents to provide future intervention targets.

**Objectives:**

During the COVID-19 pandemic, mandatory quarantine may threaten people’s psychological health and well-being. This study aimed to test the relationship between self-compassion and general well-being among self-quarantined residents and to examine the mediating role of certainty in control (i.e., a component of psychological security) in the relation. It further explored the moderated role of positive coping in the correlation between self-compassion and certainty in control.

**Methods:**

Participants were 312 self-quarantined residents (120 men, 192 women) from a community in Liaoning Province, China, who completed online questionnaires of the Self-Compassion Scale (SCS), Security Questionnaire (SQ), Simplified Coping Style Questionnaire (SCSQ), and General Well-Being Scale (GWBS). A moderated mediation model was conducted to test the hypotheses.

**Results:**

The moderated mediation model suggested that self-compassion was positively associated with well-being. Certainty in control partially mediated the relationship between self-compassion and general well-being. Moreover, positive coping moderated the relationship between self-compassion and certainty in control. The link between self-compassion and certainty in control was stronger among low-level positive coping people than high-level ones.

**Conclusions:**

Findings reveal that increased psychological security (*e.g.*, certainty in control) by self-compassion contributes to general well-being during the self-quarantined period. People with low positive coping may benefit more from self-compassion. This study thus broadens the understanding of the mechanism underlying self-compassion on positive functioning and well-being. Psychological interventions should focus on self-compassion to enhance the general well-being of quarantined people in the pandemic.

**Disclosure of Interest:**

None Declared

